# Derivation and internal validation of a mortality risk index for aged people living with HIV: The Dat'AIDS score

**DOI:** 10.1371/journal.pone.0195725

**Published:** 2018-04-19

**Authors:** Maxime Hentzien, Cyrille Delpierre, Pascal Pugliese, Clotilde Allavena, Christine Jacomet, Marc-Antoine Valantin, André Cabié, Lise Cuzin, David Rey, Firouzé Bani-Sadr, Moustapha Dramé, for the Dat’Aids Study Group

**Affiliations:** 1 Department of Internal Medicine, Infectious Diseases, and Clinical Immunology, Robert Debré Hospital, Reims Teaching Hospitals, Reims, France; 2 EA 3797, Faculty of Medicine, University of Reims Champagne-Ardenne, Reims, France; 3 UMR1027, INSERM, Université Toulouse III Paul-Sabatier, Toulouse, France; 4 Infectious Diseases Department, CHU L’Archet, University of Nice, Nice, France; 5 Infectious Diseases Department, CHU Hôtel Dieu, University of Nantes, Nantes, France; 6 Department of Infectious and Tropical Diseases, Gabriel-Montpied Hospital, Clermont-Ferrand Teaching Hospitals, Clermont-Ferrand, France; 7 Department of Infectious and Tropical Diseases, La Pitié-Salpêtrière Hospital, Paris Teaching Hospitals, Paris, France; 8 Department of Infectious and Tropical Diseases, Fort-De-France Hospital, Martinique Teaching Hospitals, Fort-De-France, Martinique, France; 9 COREVIH Toulouse, CHU Toulouse, Toulouse, France; 10 HIV Care Center, Nouvel Hôpital Civil, Hôpitaux universitaires de Strasbourg, Strasbourg, France; 11 Department of Research and Public Health, Robert Debré Hospital, Reims Teaching Hospitals, Reims, France; Azienda Ospedaliera Universitaria di Perugia, ITALY

## Abstract

**Objective:**

The objective was to develop a multivariable prognostic index for overall mortality over a five-year span integrating classical HIV biomarkers and comorbidities in people living with HIV (PLHIV) aged 60 or older.

**Design:**

Prospective multicenter cohort study from the French Dat’AIDS cohort.

**Methods:**

All HIV-1 infected patients aged 60 years or older on 1st January 2008 were included. Sociodemographic data, CD4 cell count, CD4 nadir, HIV viral load, history of comorbidities, hepatitis co-infections and laboratory parameters at baseline were considered as potential prognostic variables. Primary outcome was all-cause mortality.

**Results:**

Among 1415 patients included, we derived a score comprising the following predictors: Age (65–74: 1 point; ≥75: 8 points), CD4 cell count (200–349: 3 points; <200: 6 points), non-HIV related cancer (6 points), cardiovascular disease (8 points), estimated glomerular filtration rate (30–59 mL/min/1.73m^2^: 5 points; <30mL/min/1.73m^2^: 16 points), cirrhosis (13 points), low body mass index (<18.5 kg/m^2^, 10 points), anemia (6 points). Mean observed score was 7.0 ± 8.0 and ranged from 0 to 45. Score categories defined 4 risk groups for mortality: low, moderate, high and very high risk (5-year survival probability 0.95 (95%CI[0.93–0.97]), 0.90 (95%CI[0.87–0.92]), 0.77 (95%CI[0.68–0.84]) and 0.54 (95%CI[0.43–0.63]) respectively). The score showed good discrimination (C-statistic = 0.76) and calibration.

**Conclusions:**

We propose a multivariable prognostic score for mortality among PLHIV aged 60 or over, who will become the predominant population in future years in western populations. It could be a useful tool for research, for developing preventive and treatment strategies according to risk group, and for risk assessment by clinicians.

## Introduction

Since the advent of combination antiretroviral therapy (cART), AIDS- and non-AIDS related mortality has decreased [1,2], and HIV infection has become a long-term chronic disease. Thus, average age at infection has increased, the HIV-infected population is ageing, and the risk of late diagnosis is higher at older age [2,3]. There are several definitions of aged people living with HIV (PLHIV). The International AIDS Society previously defined aged PLHIV as aged 60 or more [4]. This definition seems adapted to western countries, where the standard geriatric age cut-off is 65 years old, while accounting for the fact that PLHIV present comorbidities earlier than similar HIV-uninfected people [5]. This new and growing aged population will be predominant in the future among PLHIV [6]. Indeed, a recent modelization study in Europe estimated that the median age of PLHIV receiving treatment will be 56.6 years in 2030, and that the proportion of patients aged 60 or older will increase from 8% in 2010 to 39% in 2030 [6]. Since it is still emerging, this population is insufficiently characterized, and identifying multivariable clinical indices, including more than just classical factors such as CD4 cell count, has become a research priority to stratify patients who are at increased risk of mortality [2,7].

Aged PLHIV have a different epidemiology compared to HIV-uninfected populations at the same age. Some data suggest that PLHIV experience accelerated aging [8], but PLHIV are also more exposed to modifiable lifestyle confounders and co-infections [9]. Comorbidity prevalence is higher, possibly with earlier onset than in the general aged population [5,10–12], although this question of earlier onset is still debated [13]. Comorbidities, especially chronic renal disease and cardiovascular diseases, are becoming increasingly determinant in aging PLHIV, with a prognostic role that is as important as HIV-related prognostic factors such as CD4 cell count [14]. The burden of comorbidities is high in this population, and will continue to increase in the future [6,15]. This different epidemiology with lifestyle confounders and higher comorbidity burden suggests that a specific score to estimate mortality risk, specifically designed for aged PLHIV, and including HIV-related factors and comorbidities, would be of major clinical and research interest [2,7].

Thus, the objective of the present study was to derive and internally validate a mortality risk index over a five-year span in a population of PLHIV aged 60 or older followed in the context of a large French cohort in the late cART era.

## Material and methods

This study involved all HIV-1 infected patients aged 60 years or older on 1st January 2008, and followed up in the context of the Dat’AIDS cohort that involves 12 French hospitals. Dat’AIDS is a French multicenter prospective cohort that covers inpatients or outpatients managed in French public hospitals, including French overseas territories. It is based on a computerized medical record that is completed by clinicians during patient visits since 2000 (Nadis®, Fedialis Medica, Marly le Roi, France)[16]. Data are collected in real time by clinicians, and not by database linkage. Diagnoses where stored in the database using International Classification of Disease, 10th Revision (ICD-10) codes. It is subject to continuous quality control, including comorbidity data. All patients were included in the cohort after receiving oral information and giving written consent. All patient information was entered into a database using anonymous, coded identification numbers. This study was performed in accordance with the principles of the Declaration of Helsinki and current French legislation relating to biomedical research. As this study relied on already existing clinical data only and as there was no intervention on study participants, there was no need of an ethics committee advice according to French laws available when the study was performed. The DatAIDS cohort is registered on Clinicaltrials.gov under the identifier NCT02898987.

Patients were excluded if they were co-infected by HIV-2 or if they did not have at least one CD4 cell count available in the 12 months before or after 1st January 2008. For each patient, follow-up began on 1st January 2008 and stopped on the date of death, or date of last follow-up, or on 1st January 2013 (whichever came first).

The study endpoint was five-year mortality. In patients lost to follow-up (defined as a date of last follow-up prior to 1st January 2013 and alive at last follow-up), vital status was systematically recorded via the Centre for Epidemiology and Population Health (CESP), by linkage with the French National Institute of Statistics and Economic Studies (INSEE), which records nearly all deaths that occur in France by centralized reception of death certificates.

The following data were collected at baseline (1st January 2008): age, gender, risk categories for HIV infection, duration of known HIV infection, AIDS status, CD4 cell count, CD4 nadir, and HIV viral load. The following comorbidities were considered at baseline, since they are known to be associated with mortality in the general population: cardiovascular disease (CVD) (history of myocardial infarction, congestive heart failure, cerebrovascular disease), cancer (non-HIV related), chronic pulmonary disease, diabetes, decreased estimated glomerular filtration rate (eGFR, defined as an eGFR below 60 mL/min/1.73m^2^, calculated using the CKD-EPI formula [17]), anemia, cirrhosis, HCV co-infection and HBV co-infection. Anemia was defined as a hemoglobin level <12g/dL for women and <13g/dL for men. HBV co-infection was defined as at least one positive hepatitis B surface antigen. HCV co-infection was defined as at least one positive anti-HCV antibody and/or detectable HCV-RNA viral load (recording the value from the assessment closest to the baseline date). Low body mass index (BMI) (<18.5 kg/m^2^) was also assessed as it is significantly associated with mortality in the general aged population[18] and in aged PLHIV[14], even after adjustment for comorbidities. Where appropriate, adapted ICD-10 coding algorithms for Charlson comorbidity index were used[19,20]. Other comorbidities were extracted using ICD-10 codes and diagnoses available before 1st January 2008.

### Statistical analysis

Mean ± standard deviation (SD) and number (percentage) were used to describe population characteristics. Univariable and multivariable analysis were performed using Cox’s proportional hazards model to generate Hazard Ratios (HR), adjusted HR (aHR) and associated 95% confidence intervals (95% CI). Quantitative variables were categorized for the purposes of the analysis. HCV co-infection was only considered for descriptive purposes, as the dramatic change in the future of the HCV epidemic may make its interpretation difficult in future years [21]. A manual, step-by-step descending selection of covariates was used. Covariates with a p-value >0.20 were excluded. Hazard ratio significance was determined using the global p-value of the two-sided Wald test. Significance was reached when p<0.05. We also performed bootstrap analysis to evaluate the internal validity of the model performance. Bootstrap analysis is the preferred method for internal validation, especially when the development sample is relatively small and/or a high number of candidate predictors is studied [22]. Replication on 2000 different samples drawn with replacement was performed for the bootstrap method. The C-statistic of the model and the May & Hosmer test for goodness-of-fit [23] were used to assess model discrimination and calibration.

For the development of the score, a point value was assigned to each independent factor according to the parameter estimates of the final model. Parameter estimates were multiplied by 10, rounded to the nearest integer and summed. The population was then separated into four risk groups according to the score: low-risk, moderate-risk, high-risk, very-high-risk. The C-statistic was also calculated. Log-linearity and the proportional hazards assumptions were also checked for the score thus obtained. Baseline survival probability was modelled using quadratic regression. Survival probability was then generated using Cox’s proportional hazards model formula. Predicted (Cox’s model) versus observed (Kaplan Meier estimates) survival was then plotted to assess the score calibration and discrimination.

Statistical analyses were performed using SAS version 9.4 (SAS Institute Inc., Cary, North Carolina, USA).

## Results

Among 18,304 HIV-1 infected individuals actively followed up as of 1st January 2008, 1583 (8.6%) were aged 60 or older. Among these, 168 were excluded due to missing data for CD4 cell count within one year before or after baseline, and 1,415 patients (89.4%) were thus included in the final analysis. The baseline characteristics of the study population are presented in Table 1. Most patients were male (77.2%) and mean age was 65.7±5.5 years. Duration of known HIV infection was 11.9 ±6.1 years and 965 (68.2%) patients were diagnosed at age 50 or older. Main comorbidities were anemia (21.4%), decreased eGFR (21.1%), diabetes (14.2%), and cardiovascular diseases (12.2%). Among patients with eGFR <60 ml/min/1.73m^2^, mean eGFR was 48.0±11.5 ml/min/1.73m^2^.

**Table 1:** Baseline characteristics of the 1,415 PLHIV aged 60 years or more from the Dat’AIDS cohort.

	Baseline characteristics (N = 1415)	
Male sex [n (%)]	1093	(77.2)
Age (years) [mean (±SD)]	65.7	(±5.5)
60–64 years [n (%)]	754	(53.3)
65–74 years [n (%)]	544	(38.5)
75 years or more [n (%)]	117	(8.3)
Mode of HIV infection [n (%)]		
Heterosexual	630	(44.5)
Homosexual	556	(39.3)
Injecting drug user	9	(0.6)
Other	220	(15.6)
Duration of known HIV infection (years) [mean (±SD)]	11.9	(±6.1)
ART-experienced [n (%)]	1248	(88.2)
Duration of ART treatment (years) [mean (±SD)] (n = 1248)	9.6	(±4.8)
AIDS [n (%)]	426	(30.1)
Age-related comorbidities [n (%)]		
Cancer	229	(16.2)
*Non HIV-related cancer*	94	(6.6)
Cardiovascular diseases	172	(12.2)
*Cerebrovascular disease*	77	(5.4)
*Myocardial infarction*	67	(4.7)
*Congestive heart failure*	42	(3.0)
Chronic pulmonary disease	112	(7.9)
Decreased eGFR	285	(20.1)
Diabetes	201	(14.2)
Cirrhosis	39	(2.8)
HBV co-infection	54	(3.8)
HCV co-infection	92	(6.5)
Low body mass index (<18.5 kg/m^2^) (missing = 29) [n (%)]	76	(5.5)
CD4 cell count (cells/μl) [mean (±SD)]	507	(±245)
>500 cells/μl	653	(46.2)
350–500 cells/μl	364	(25.7)
200–349 cells/μl	299	(21.1)
<200 cells/μl	99	(7.0)
CD4 nadir (cells/μl) [mean (±SD)]	210	(±174)
≥200 cells/μl [n(%)]	619	(43.8)
<200 cells/μl [n(%)]	796	(56.2)
HIV Viral Load >50 copies/ml [n (%)] (missing = 3)	331	(23.4)
Estimated glomerular filtration rate (CKD-EPI) (missing = 28)		
≥60 ml/mn/1.73m^2^ [n(%)]	1095	(78.9)
30–59 ml/mn/1.73m^2^ [n(%)]	266	(19.2)
<30 ml/mn/1.73m^2^ [n(%)]	26	(1.9)
Anemia* (missing = 33)	296	(21.4)

PLHIV, people living with HIV; HIV, human immunodeficiency virus; AIDS, acquired immune deficiency syndrome; ART, antiretroviral treatment; HBV, hepatitis B virus; HCV, hepatitis C virus; eGFR, estimated glomerular filtration rate; SD, standard deviation

*Anemia was defined as a hemoglobin level <12g/dL for women, and <13g/dL for men

During the five years of follow-up (6,225 patient-years), 154 (10.9%) patients died. Mean age at death was 70.5 ± 7.4 years. One thousand and fifty-three surviving PLHIV (74.4%) were fully followed until the end of the study period, and 208 (14.7%) patients were lost to follow-up (median follow up among these patients was 2.3 years [2.0–3.4]).

By univariable analysis (Table 2), factors associated with overall five-year mortality were age, AIDS status, non-HIV related cancer, cardiovascular disease, decreased eGFR, cirrhosis, anemia, low BMI, CD4 cell count, CD4 nadir, and detectable HIV viral load.

**Table 2:** Factors associated with overall 5-year mortality among PLHIV aged 60 years or over by univariable and multivariable analysis (N = 1415).

	Univariable analysis			Multivariable analysis (n = 1366)		
	HR	95% CI	P value	aHR	95%CI	P value
Age (years)						
60–64	-	-	-	-	-	-
65–74	1.25	[0.88–1.77]	0.22	1.07	[0.74–1.54]	0.73
≥ 75	3.06	[1.97–4.76]	<10^−4^	2.17	[1.35–3.50]	0.001
Male sex	0.91	[0.63–1.31]	0.60			
AIDS	1.51	[1.09–2.09]	0.01			
Non-HIV related cancer	2.61	[1.66–4.09]	<10^−4^	1.91	[1.19–3.05]	0.007
Cardiovascular disease	3.23	[2.28–4.57]	<10^−4^	2.24	[1.55–3.23]	<10^−4^
Estimated glomerular filtration rate (CKD-EPI))						
≥60 ml/mn/1.73m^2^ [n(%)]	-	-	-	-	-	-
30–59 ml/mn/1.73m^2^ [n(%)]	2.22	[1.56–3.16]	<10^−4^	1.64	[1.13–2.37]	0.01
<30 ml/mn/1.73m^2^ [n(%)]	8.58	[4.80–15.36]	<10^−4^	5.18	[2.79–9.60]	<10^−4^
Chronic pulmonary disease	1.54	[0.94–2.53]	0.08			
Diabetes	1.43	[0.96–2.14]	0.08			
Cirrhosis	4.41	[2.59–7.51]	<10^−4^	3.63	[2.10–6.30]	<10^−4^
HBV co-infection	0.80	[0.33–1.95]	0.62			
Low body mass index (<18.5 kg/m^2^)	3.78	[2.42–5.89]	<10^−4^	2.60	[1.65–4.09]	<10^−4^
CD4 cell count (cells/μl)						
>500	-	-	-	-	-	-
350–500	1.12	[0.73–1.71]	0.60	0.91	[0.58–1.41]	0.67
200–349	1.73	[1.16–2.58]	0.007	1.32	[0.87–2.00]	0.20
<200	2.88	[1.74–4.76]	<10^−4^	1.85	[1.09–3.16]	0.02
CD4 nadir (cells/μl), ≤200	1.55	[1.11–2.17]	0.01			
HIV viral load (copies/ml), >50	1.47	[1.04–2.08]	0.03			
Anemia*, yes	3.02	[2.19–4.17]	<10^−4^	1.89	[1.33–2.68]	0.0003

95% CI, 95% confidence interval; HR, Hazard Ratio; aHR, Adjusted Hazard Ratio; HIV, human immunodeficiency virus; AIDS, acquired immune deficiency syndrome; HBV, hepatitis B virus.

*Anemia was defined as a hemoglobin level <12g/dL for women, and <13g/dL for men.

Bootstrapping showed excellent internal validity.

The May & Hosmer goodness of fit test did not identify calibration issues (p>0.80 for each stratum)

Multivariable model C-statistic = 0.75 (95%CI [0.71–0.79])

By multivariable analysis (Table 2), factors that remained significantly associated with mortality were age, non-HIV related cancer, cardiovascular disease, decreased eGFR, cirrhosis, anemia, low BMI, and CD4 cell count. Bootstrapping of the multivariable model showed excellent internal validity. The May & Hosmer test did not identify any calibration issues (p>0.80 for each stratum) and the model’s C-statistic was 0.75 (95%CI [0.71–0.79]).

Using parameter estimates, a mortality risk index was derived from the multivariable model (Table 3). The score theoretically ranged from 0 to 73. Mean observed score was 7.0 ± 8.0 and ranged from 0 to 45. It met the assumptions of log-linearity and proportional hazards. A survival equation was then derived from the baseline survival equation and parameter estimates for each one-point increase of the score (Table 3).

**Table 3:** Point value assigned to each predictor of 5-year overall mortality.

Predictive factor	Point value
Age	
60–64 years old	0
65–74 years old	1
≥75 years old	8
CD4 cell count	
>500/mm^3^	0
350–500/mm^3^	0
200–349/mm^3^	3
<200/mm^3^	6
Non-HIV related cancer	6
Cardiovascular disease	8
Estimated glomerular filtration rate (CKD–EPI formula)	
≥60 ml/mn/1.73m^2^	0
30–59 ml/mn/1.73m^2^	5
<30 ml/mn/1.73m^2^	16
Cirrhosis	13
Low BMI	10
Anemia*	6

BMI: Body mass index

*Anemia was defined as a hemoglobin level <12g/dL for women, and <13g/dL for men.

Score’s C-statistic = 0.76 (95% CI [0.72–0.80]).

Log linearity and proportional hazards assumptions were verified.

For the development of the score, parameter estimates were multiplied by 10 and rounded to the nearest integer (e.g., for 65–74 years old the score of 1 is simply round(10*log(1.07)) and summed.

Survival prediction formula after modelization of baseline survival (S_0_(t)) by quadratic regression

$$S(t)={\left(1-0.00562*time(years)-0.00057679*time^{2}(years)\right)}^{e^{score*0.1008354015}}$$

For example, a patient with a score equal to 10 will have a calculated 2-year survival probability of 96% and a calculated 5-year survival probability of 89%.

**Fig 1:**
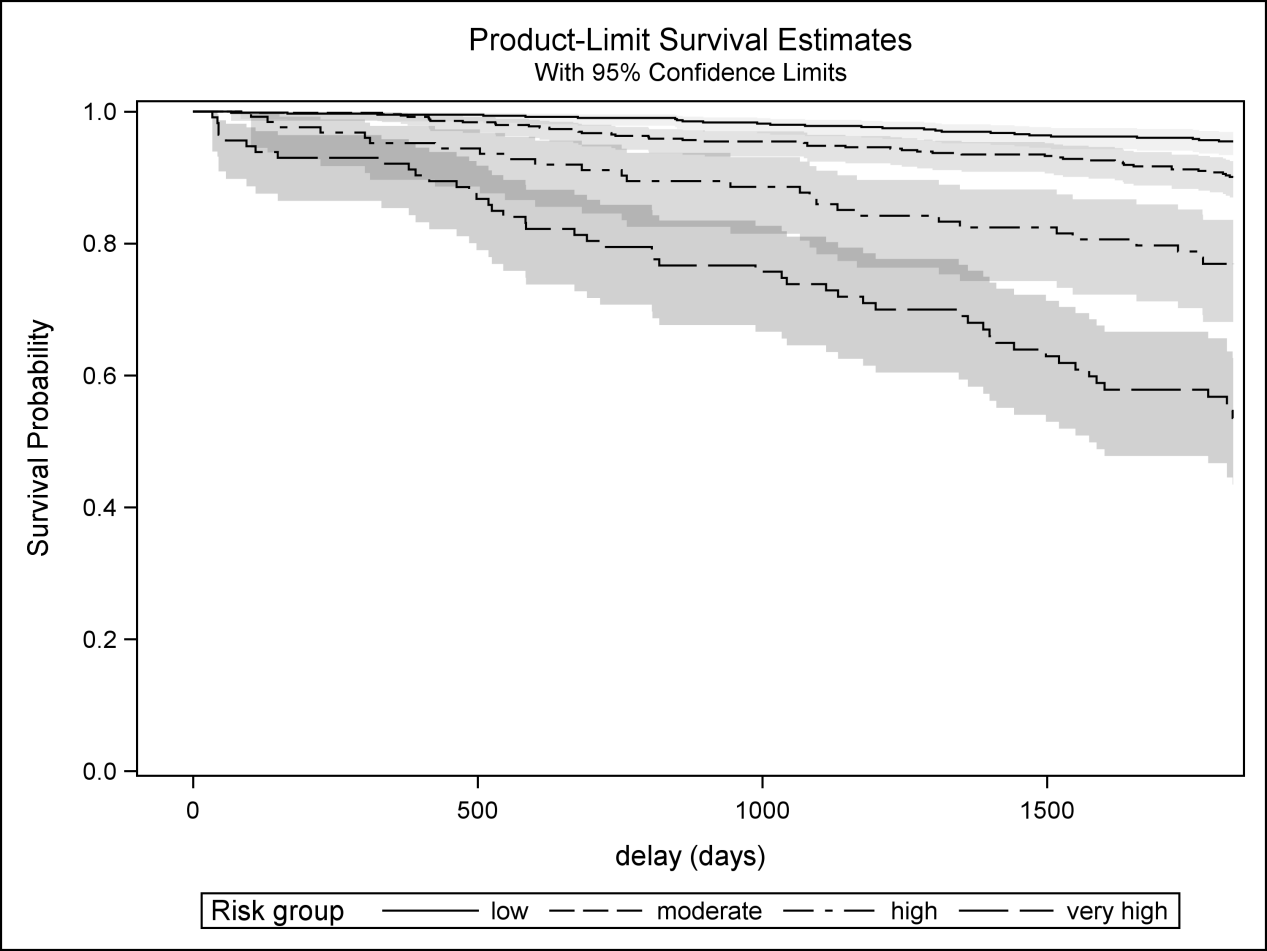
Five-year Kaplan-Meier survival probabilities among each risk group.

**Fig 2:**
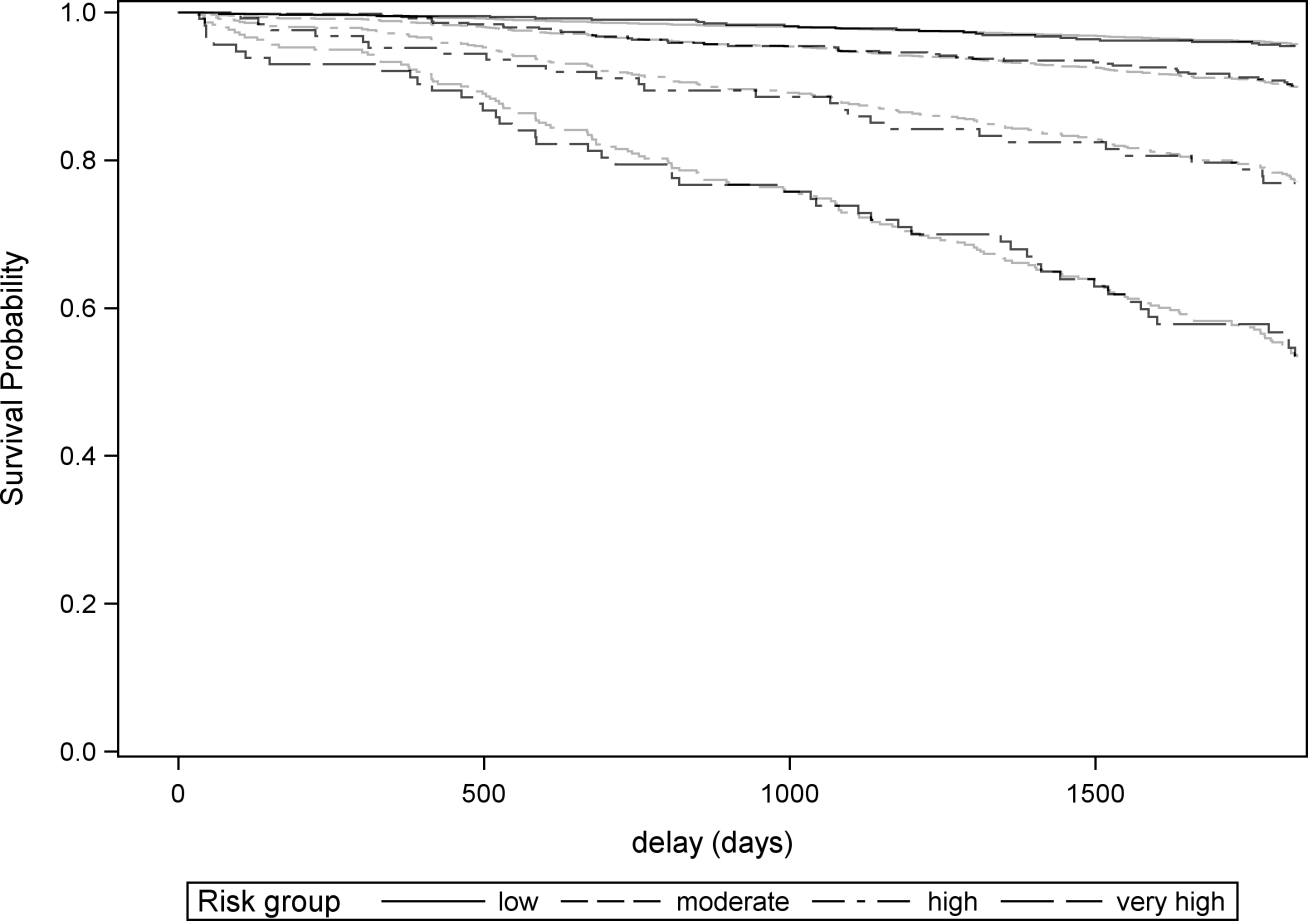
Assessment of model calibration: Observed (Kaplan Meier, black) versus predicted (Cox, gray) survival curves.

Five-year survival probabilities for each risk group according to the Kaplan-Meier estimates and the baseline survival equation are presented in Table 4 and Fig 1. Each increase of one risk-group resulted in a significant 2 to 2.5-fold multiplication of the hazard ratio (Table 5), indicating good discrimination. Plotting observed versus predicted survival curve for each risk group showed excellent calibration (Fig 2).

**Table 4:** Five-year Kaplan-Meier survival probabilities in each score group.

Risk group	Score value	Number of patients	Five-year survival probability
Low risk	0–3	626	0.95 [0.93–0.97]
Moderate risk	4–13	500	0.90 [0.87–0.92]
High risk	14–20	126	0.77 [0.68–0.84]
Very high risk	≥20	114	0.54 [0.43–0.63]
Baseline survival probability formula for low risk group after modelization by quadratic regression: S_0_(t) = 1–0.00610*time_(years)_ - 0.00050468*time_(years)_^2^			

Baseline survival probability formula for low risk group after modelization by quadratic regression

S_0_(t) = 1–0.00610*time_(years)_ - 0.00050468*time_(years)_^2^

**Table 5:** Model discrimination: Hazard ratio across risk groups.

Risk group	Hazard ratio	95% CI
Moderate vs. low risk	2.27	[1.40–3.67]
High vs. moderate risk	2.56	[1.59–4.12]
Very-High vs. High	2.36	[1.48–3.78]

## Discussion

A specific predictive score for aged PLHIV is of primary interest for the near future. Aged PLHIV will be predominant in the future of the epidemic[6]. To date, there is no specific index available to stratify mortality risk among aged HIV populations. From this large representative cohort of aged French comorbid PLHIV followed up for five years in the late cART era, we developed a multivariable comorbidity index that enables accurate prediction of all-cause mortality over a 5-year period. The Dat’AIDS score comprises simple and reliable predictors such as age, CD4 cell count, history of non-HIV related cancer, history of cardiovascular disease, eGFR, cirrhosis, BMI and anemia, which are all easily obtainable at assessment.

We showed that most of our patients were diagnosed at older ages. It was showed that patients aging with HIV tended to have more multimorbidity and may have different comorbidity profiles comparatively to patients diagnosed at an older age [24]. The part of patients aging with HIV will likely increase in comparison with patients HIV-aged, suggesting that the prevalence of comorbidities at similar ages will increase in future years. To date, it is unknown whether the impact of comorbidities is different between these two populations. However, it is possible that the role of some comorbidities, especially those related in part to the long-term cART toxicities as renal and cardiovascular diseases, may differ as they tend to have different metabolic profiles [14,24,25]. More research is needed on this point.

The good immunovirological control may explain the marginal role of CD4 cell count and viral load with regard to comorbidities, and explain that the main prognostic criteria for this score were age, eGFR, cardiovascular disease, cirrhosis and low BMI. The role of those comorbidities in the mortality of aged PLHIV raises important issues in the long-term management of these patients, as the cumulative use of tenofovir is associated with the onset of chronic renal disease, especially when associated with protease inhibitors [25,26], and as the cumulative use of protease inhibitors is associated with cardiovascular diseases [27].

The score showed good discriminatory capacity and model adequacy was very good. It uses comorbidities as prognostic items, as these are concrete life events with a major impact that may not be sensitive to change in acute situations, thus allowing the clinician to evaluate the background prognosis even in the acute context. The score was developed in a sample representative of the late cART era, which is more adapted to the future aged population, and makes it possible to define 4 risk groups for mortality, namely low, moderate, high and very high risk (5-year survival probabilities 0.95, 0.90, 0.77 and 0.54 respectively).

Other mortality indices such as the VACS index[28,29], which accounts for organ injuries (including hemoglobin, FIB-4 score and eGFR) and classical HIV biomarkers (including CD4 cell-count and HIV-1 RNA) but not comorbidities (except eGFR), have not been specifically evaluated or validated in an aged population over 60, as only 242 patients were aged 65 or older in the development study [28], and 53 [28] and 162 [29] were aged >65 years in the validation studies, compared to 661 patients aged over 65 in the present study.

The score presented here could be useful when assessing individual risk-benefit ratios, by improving the accuracy of mortality risk assessment, or to define populations that may benefit the most from intervention. Such a score may reflect the extra burden of multimorbidity and polypharmacy in the aged PLHIV population [2,30] and could be associated with frailty measures [31–33].

In the research field, we believe the present score may be a useful tool to stratify patients for risk of death in observational or intervention studies, or as an adjustment variable, just as the Charlson comorbidity index is in general population [20], the Framingham score [25] for cardiovascular risk or Fried’s score[26] for frailty. Future treatment optimization strategies according to the underlying health status are likely as in the geriatric population. The Dat’AIDS score could also be a useful tool to develop such strategies according to risk groups.

Some limitations of the present work deserve to be acknowledged. Certain other comorbidities might also be related to the outcome and warrant being added to the present score. For example, dementia and cognitive impairment, which may be more prevalent in PLHIV [34], would be good candidate variables. However, patients did not have systematic geriatric assessment, likely leading to underestimation of its prevalence in the cohort. Accounting for HCV co-infection may have strengthened the predictive ability of the present score [14], however the dramatic change looming in the future of the HCV epidemic [21,35] may make its interpretation difficult in future years in countries with access to direct-acting antivirals. Some comorbidities may not respect the proportional hazards assumption, as we previously showed for chronic renal disease and low BMI [14]. However, the present score does support the assumption of proportional hazards. Another limit to be acknowledged is that the 5-year survival probability equation for the score used as a continuous variable may not be adequate for scores over 30–35. Indeed, although the observed score ranged up to 45, such scores were very infrequent in our study population, and a ceiling effect at such scores is likely, leading to poor estimation of survival probability at high values. Moreover, the proposed equation should not be used for risk prediction beyond five years, as it was not designed for this purpose, and data for longer time periods were not available to enable accurate baseline survival modelization over periods exceeding five years. The present score could not be considered as a frailty index, as it comprises age and considers a limited number of deficits. Finally, and most importantly, external validation in another population is mandatory before recommending the wider use of this score among aged PLHIV. Baseline survival probability formula was modelized in order to generate individual survival probabilities and to allow for calibration when external validation is performed [36]. During external validation, the present score should be compared with other prognostic scores such as the VACS index or the updated Charlson comorbidity index [28,29]. As the Dat’AIDS score was derived from the present data, it was not possible to directly compare it with other prognostic scores since it would have favored it. The present score could also be tested in PLHIV aged 50 or older, as the comorbidity prevalence remains high in this population.

## Conclusions

We propose the first multivariable prognostic score for mortality specific to PLHIV aged 60 or over and developed in the late cART era. The score showed good discrimination and calibration and may be, once externally validated, a useful tool for research as well as for risk assessment by clinicians, especially since the population of aged PLHIV will become increasingly predominant in future years.
